# Comparison of Inter Subject Variability and Reproducibility of Whole Brain Proton Spectroscopy

**DOI:** 10.1371/journal.pone.0115304

**Published:** 2014-12-17

**Authors:** Tonny V. Veenith, Marius Mada, Eleanor Carter, Julia Grossac, Virginia Newcombe, Joanne Outtrim, Victoria Lupson, Sridhar Nallapareddy, Guy B. Williams, Sulaiman Sheriff, David K. Menon, Andrew A. Maudsley, Jonathan P. Coles

**Affiliations:** 1 Department of Critical Care Medicine, University hospitals of Birmingham NHS trust, Queen Elizabeth Hospital, Edgbaston, Birmingham, United Kingdom, B15 2TH; 2 Division of Anaesthesia, University of Cambridge, Addenbrooke's Hospital, Hills Road, Cambridge, Cambridgeshire, United Kingdom, CB2 0QQ; 3 Wolfson Brain Imaging Centre, Department of Clinical Neurosciences, University of Cambridge, Addenbrooke's Hospital, Cambridge, United Kingdom, CB2 0QQ; 4 Department of Radiology, Miller School of Medicine, University of Miami, Miami, Florida, United States of America; Brown University, United States of America

## Abstract

The aim of these studies was to provide reference data on intersubject variability and reproducibility of metabolite ratios for Choline/Creatine (Cho/Cr), N-acetyl aspartate/Choline (NAA/Cho) and N-acetyl aspartate/Creatine (NAA/Cr), and individual signal-intensity normalised metabolite concentrations of NAA, Cho and Cr. Healthy volunteers underwent imaging on two occasions using the same 3T Siemens Verio magnetic resonance scanner. At each session two identical Metabolic Imaging and Data Acquisition Software (MIDAS) sequences were obtained along with standard structural imaging. Metabolite maps were created and regions of interest applied in normalised space. The baseline data from all 32 volunteers were used to calculate the intersubject variability, while within session and between session reproducibility were calculated from all the available data. The reproducibility of measurements were used to calculate the overall and within session 95% prediction interval for zero change. The within and between session reproducibility data were lower than the values for intersubject variability, and were variable across the different brain regions. The within and between session reproducibility measurements were similar for Cho/Cr, NAA/Choline, Cho and Cr (11.8%, 11.4%, 14.3 and 10.6% *vs.* 11.9%, 11.4%, 13.5% and 10.5% respectively), but for NAA/Creatine and NAA between session reproducibility was lower (9.3% and 9.1% *vs.* 10.1% and 9.9%; p <0.05). This study provides additional reference data that can be utilised in interventional studies to quantify change within a single imaging session, or to assess the significance of change in longitudinal studies of brain injury and disease.

## Introduction

Proton magnetic resonance spectroscopic imaging (^1^HMRS) can be used in the diagnosis, assessment of progression and prediction of outcome in a variety of neurological disorders such as brain tumours [1], traumatic brain injury [2]–[4], multiple sclerosis [5], [6], motor neuron disease [7], Alzheimer's dementia [8] and psychiatric disorders [9]–[11]. The metabolites reliably measured with proton spectroscopy (^1^HMRS) at medium to long echo times include N-acetyl aspartate (NAA), Creatine (Cr) and Choline (Cho) containing compounds. These provide a measure of neuronal integrity, metabolism and a marker of neuronal breakdown and turnover respectively [12]–[15]. While targeted imaging of regions of interest (with single voxel or two dimensional ^1^HMRS) allows evaluation of local neuronal loss and glial proliferation, whole brain imaging provides assessment of the global burden of neurological disease even in regions that appears structurally normal. ^1^HMRS has been used to non-invasively evaluate normal appearing brain in a variety of neurological disorders including multiple sclerosis and head injury [3], [6], [16]. Whole brain proton spectroscopy (WB ^1^HMRS) data acquired with Metabolic Imaging and Data Acquisition Software (MIDAS) [17]–[19] provides a fully automated pipeline for processing and interpreting WB ^1^HMRS data. Previous studies using MIDAS and other ^1^HMRS techniques have provided invaluable reference data regarding normal values within different brain regions and reproducibility of such data [20]–[24]. However, there are limited data comparing intersubject variability and reproducibility of WB ^1^HMRS measurements within the same imaging session (within session reproducibility) and those obtained during repeat imaging sessions on different days (between session reproducibility). This is of relevance for group comparisons with healthy controls, and longitudinal and interventional studies where WB ^1^HMRS is used as a biomarker of disease progression or response to therapy. The rational design and interpretation of such studies is hampered by lack of knowledge regarding how the variability of WB ^1^HMRS measurements in data obtained during the same scanning session differs when compared with similar data obtained during a different session or day. In studies where consecutive measurements are performed on each subject under resting and experimental conditions problems associated with variation between subjects due to individual differences (intersubject variability) can be limited. However, baseline MIDAS WB ^1^HMRS measurements may vary within an individual patient (intrasubject variability) and limit the ability to detect significant changes over time or following a therapeutic intervention. Where imaging is repeated after several days or weeks in different sessions the measurements may vary within an individual patient even in the absence of disease progression due to a combination of intrasubject and scanner variability [25]. Without knowledge of such differences it is difficult to accurately determine the clinical significance of pathophysiological changes, as they evolve following various causes of brain injury or disease.

The aim of these studies was to provide reference data on intersubject variability and reproducibility of commonly used metabolite ratios (Cho/Cr, NAA/Cho and NAA/Cr) and individual signal-intensity normalised metabolite concentrations (NAA, Cho and Cr) in a group of healthy volunteers using MIDAS. These data will inform the design of interventional studies, where repeated measurements are conducted within the same session, and longitudinal studies where assessments are repeated over time in several different imaging sessions.

## Materials and Methods

### Ethics statement

Ethical approval was obtained from the Cambridgeshire 2 Research Ethics Committee (reference number 97/290), and written informed consent was obtained from all volunteers in accordance with the Declaration of Helsinki.

### Imaging data acquisition

Thirty two healthy volunteers without any history of neuropsychiatric disorder or substance abuse underwent imaging using a 3T Siemens Verio MRI scanner (Siemens AG, Erlangen, Germany) with 12 channel detection within the Wolfson Brain Imaging Centre (WBIC), University of Cambridge. All volunteers were right handed (fourteen males and eighteen females) with mean (range) age of 34 (25 – 50) years, and were employed by Cambridge University Hospitals NHS Trust. Twenty-two volunteers attended a second imaging session within a mean (range) of 33 (3 – 181) days. At each imaging session subjects were imaged twice with MIDAS along with standard structural imaging. Structural sequences included 3D T1-weighted magnetisation prepared rapid gradient echo (MPRAGE), fluid attenuated inversion recovery (FLAIR), gradient echo and dual spin echo (proton density/T2-weighted). Whole brain spectroscopy data were acquired using a volumetric spin echo (TR/TE 1710/70 milliseconds, flip angle of 73°, 50 phase encoding steps and a field of view of 280×280×180 mm^3^) covering the whole brain with an acquisition time of 26 minutes as described by Maudsley et al [17], [18], [26]. This sequence also included lipid inversion nulling and an unsuppressed water spectroscopy dataset acquired with 20° flip angle acquired in an interleaved fashion. The MIDAS and MPRAGE were acquired at an angulation of ^+^15 to ^+^20° to the AC-PC line to improve brain coverage and limit field inhomogenieties from the frontal and sphenoid air sinuses. The MPRAGE (TR/TE 2150/4.4 and flip angle 8°) was acquired within each imaging session with one millimetre isotropic resolution.

### Spectroscopic data processing

**Figure 1:**
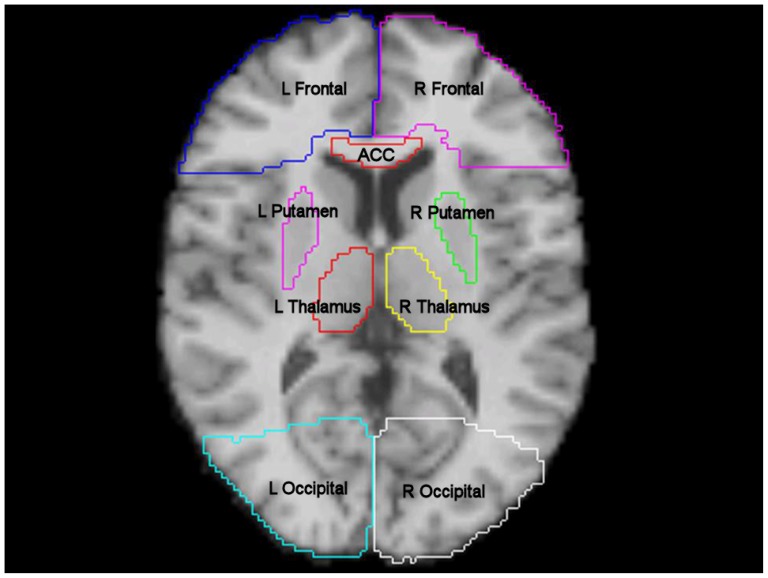
Region of interest template. T1 weighted magnetic resonance image in MNI152 space (2mm resolution) showing frontal lobe left (L Frontal), frontal lobe right (R Frontal), anterior corpus callosum (ACC), thalamus left (L Thalamus), thalamus right (R Thalamus), occipital left (L Occipital), occipital right (R Occipital), putamen left (L Putamen) and putamen right (R Putamen). Additional regions not shown include body corpus callosum, posterior corpus callosum, dorsal mid brain, ventral mid brain and bilateral regions covering the corticospinal tract, anterior thalamic radiation, inferior longitudinal fasciculus, superior longitudinal fasciculus, pallidum, hippocampus, parietal lobe, temporal lobe, cerebral peduncle and pons.

Parametric maps were created using the automated pipeline of MIDAS and the data for NAA, Cho and Cr were individual signal-intensity normalised to institutional units (iu) based on the tissue water signal derived from the water reference dataset. Metabolite data were reconstructed using MIDAS and resulted in images composed of 64×64×32 voxels with an individual voxel volume of approximately 1 ml. Voxel data with line width greater than 12 Hz were excluded from further analysis as previously described by Maudsley et al [24]. The WB ^1^HMRS parametric maps were spatially normalised using a two-step approach using FSL [20], [27]. First, control T1 weighted images were coregistered to water spectroscopic images using FMRIB's Linear Image Registration Tool (FLIRT) [28]–[30]. This was followed by coregistration of control T1 weighted images to the MNI152 template using FMRIB's Non-linear Image Registration Tool (FNIRT) [29]–[31]. Combined transformation matrixes were then applied to all parametric images used in the analyses. Representative white matter, deep grey and mixed regions of interest (ROIs) from the Harvard Oxford subcortical and MNI structural probabilistic atlases available within FSL were then applied in normalised space (Fig. 1). All coregistered images were subsequently inspected to ensure that the ROIs were correctly aligned and corresponded to the regions specified. The ROI template was modified by erosion of a single voxel using FSL to improve spatial localisation and reduce the impact of coregistration, normalisation and partial volume errors. The mean values for metabolites for each ROI were calculated using in-house software written in Matlab (Mathworks, Natick, USA).

### Analysis Strategy

Each of the 32 volunteers were invited to attend two separate imaging sessions where two MIDAS sequences were obtained. This resulted in a maximum of four independent sets of WB ^1^HMRS data (runs) for each subject, which could be used to assess the reproducibility of measurements. Twenty-two subjects underwent imaging in both sessions. The baseline data from all 32 volunteers were used to calculate intersubject variability. For the repeat MIDAS measurements obtained in the same subject the data were split into that obtained during the same imaging session and that obtained in two different imaging sessions to calculate within session and between session reproducibility respectively. Therefore, the available paired data from each session (run 1 & 2 and 3 & 4 respectively) were used to calculate within session reproducibility, and the available combinations of the four datasets from the different sessions were used to calculate between session reproducibility (runs 1 & 3, 1 & 4, 2 & 3, and 2 & 4). The inclusion of all potential combinations ensures that any variation in the order of the individual sequences obtained within each particular session is accounted for within the calculated average measurement of between session reproducibility and reflects clinical practice.

In order to help design any future interventional study using proton spectroscopy we need to know how much deviation in a repeat measurement we would accept as no or zero change. We used the SD of measurements obtained in this healthy volunteer study to calculate a ‘confidence interval’ for zero change of a repeat measurement in the same subject. We used the average SD for all measurements obtained in 32 volunteers in both sessions to calculate the population 95% prediction interval (PI) for zero change (using two SD values) [25], [32], [33]. These calculated thresholds are prediction intervals for assuming no changes from zero with the repeat WB ^1^HMRS measurement rather than confidence intervals for variability of the measurement. This estimate for the variation in repeat measurements means that we would accept a positive or negative change in a patient as being indicative of zero change as long as it were less than 2 times the standard deviation of the repeat measurement obtained in our healthy volunteer group. Although these average data are extremely useful, the calculated SD could vary within different sessions and particular ROIs within subjects. It would therefore be helpful to have a more specific measure of variability within a session (within session reproducibility), and preferably for each ROI. While this is possible, the small sample numbers (two readings obtained in each of the two sessions) means that a conventional threshold of change greater than 2SD cannot be used to assess the statistical significance of changes in this context. For a *t* distribution with two degrees of freedom, statistical theory suggests that an estimate of the 95% prediction interval for zero change may be provided by a threshold of 4.3 SDs. These within session measurements could therefore be used to assess the significance of the changes in WB ^1^HMRS parameters following a therapeutic intervention within the same imaging session. We have previously published this analysis strategy for diffusion tensor imaging and ^15^O positron emission tomography [25], [34].

### Statistical analysis

Statistical analyses were conducted using Statview (Version 5, 1998, SAS Institute Inc., Cary, North Carolina, USA) and SPSS Statistics Version 21 (IBM Corporation, New York, United States). All data are expressed and displayed as mean and standard deviation (SD), unless otherwise stated. To compare the reproducibility of WB ^1^HMRS measurements the SD and coefficient of variation (CoV) (CoV  =  SD/mean) of measurements were calculated within each ROI. Data were compared using paired t-tests, factorial analysis of variance (ANOVA) and intraclass correlation (ICC) as appropriate. Using ANOVA the residual standard deviation was used to calculate the 95% prediction interval for zero change of repeat WB ^1^HMRS studies. All *p* values are quoted after Bonferroni corrections for multiple comparisons (where appropriate).

## Results

### Intersubject variability for whole brain proton spectroscopic imaging (WB ^1^HMRS)

**Figure 2:**
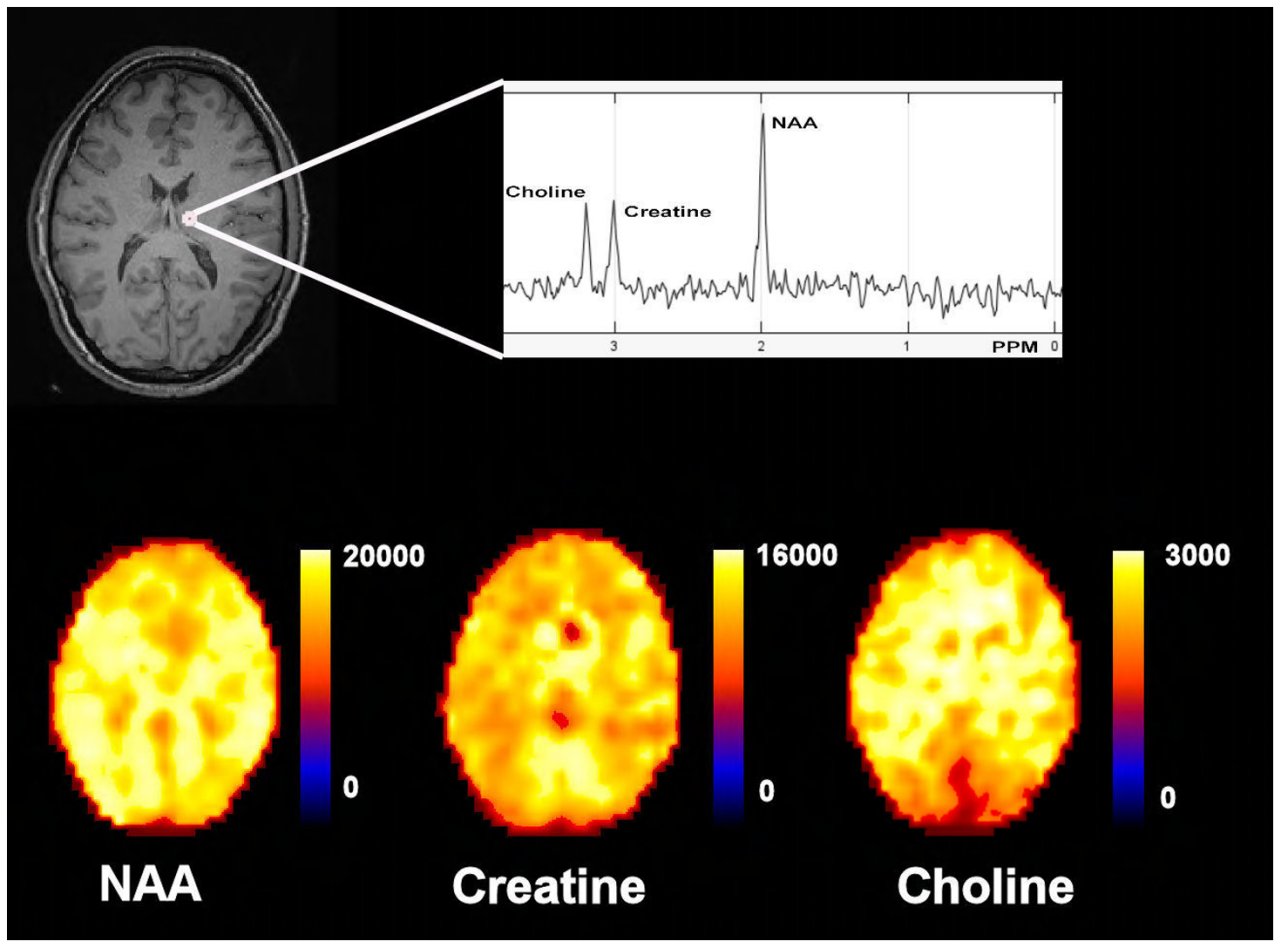
Metabolite parametric maps. T1 weighted magnetic resonance image in MNI152 space (2mm resolution) with a representative spectra from the right thalamus and N-acetyl aspartate (NAA), Creatine (Cr) and Choline (Cho) signal-intensity normalised metabolite concentration parametric maps. PPM (parts per million).

The intersubject variability of the metabolite ratios (Cho/Cr, NAA/Cr and NAA/Cho) and concentrations (NAA, Cho and Cr) using the ROI template (Fig. 1) are displayed in Table 1 and 2 respectively. In Fig. 2 NAA, Cr and Cho signal-intensity normalised metabolite concentration parametric maps are displayed in comparison with a structural image. The intersubject variability was high with a mean (range) CoV across the ROIs for Cho/Cr of 21 (11 – 62%), NAA/Cho 17 (11 – 55%), NAA/Cr 13 (8 – 37%), NAA 12 (6 – 23%), choline of 31 (13 – 69%) and creatine 19 (7 – 61%).

**Table 1:** Intersubject variability of metabolite ratios for whole brain proton spectroscopy.

	Choline/Creatine			NAA/Choline			NAA/Creatine		
Region of Interest	Mean	SD	CoV %	Mean	SD	CoV %	Mean	SD	CoV %
Anterior corpus callosum	0.63	0.39	61.81	5.23	2.85	54.58	1.79	0.67	37.35
Body of corpus callosum	0.27	0.07	24.47	6.45	0.93	14.47	1.59	0.23	14.35
Posterior corpus callosum	0.28	0.05	17.04	6.78	0.78	11.53	1.81	0.23	12.86
Corticospinal tract right	0.29	0.04	13.57	4.98	0.67	13.41	1.40	0.14	10.26
Corticospinal tract left	0.29	0.06	21.11	4.85	0.56	11.57	1.37	0.12	8.62
Anterior thalamic radiation right	0.32	0.06	18.37	5.29	0.67	12.68	1.44	0.12	8.30
Anterior thalamic radiation left	0.31	0.07	22.61	5.54	0.68	12.26	1.46	0.11	7.63
Inferior longitudinal fasciculus right	0.24	0.04	17.26	7.16	0.81	11.35	1.49	0.12	8.09
Inferior longitudinal fasciculus left	0.22	0.03	13.13	6.65	0.78	11.68	1.38	0.12	8.46
Superior longitudinal fasciculus right	0.23	0.03	11.93	6.77	0.83	12.30	1.50	0.13	8.57
Superior longitudinal fasciculus left	0.21	0.03	12.51	6.26	0.78	12.52	1.36	0.11	8.17
Thalamus right	0.31	0.05	15.84	5.37	0.68	12.58	1.59	0.18	11.57
Thalamus left	0.30	0.05	15.62	5.58	0.67	12.08	1.63	0.20	12.09
Pallidum right	0.29	0.05	16.50	5.71	0.84	14.67	1.49	0.12	8.23
Pallidum left	0.30	0.07	22.54	5.82	0.79	13.66	1.54	0.16	10.18
Putamen right	0.28	0.04	15.62	5.75	0.84	14.65	1.47	0.14	9.88
Putamen left	0.30	0.06	20.25	5.70	0.74	12.95	1.50	0.15	10.18
Dorsal Mid Brain	0.37	0.08	21.14	4.84	0.94	19.49	1.71	0.22	13.10
Ventral Midbrain	0.35	0.08	21.93	5.11	0.78	15.34	1.64	0.14	8.33
Frontal lobe right	0.21	0.06	27.24	4.42	0.87	19.79	0.97	0.13	13.79
Frontal lobe left	0.22	0.09	40.53	4.25	0.90	21.13	0.92	0.14	15.03
Hippocampus right	0.33	0.06	16.57	4.97	0.95	19.14	1.49	0.21	13.87
Hippocampus left	0.33	0.04	13.19	4.88	0.91	18.59	1.50	0.23	15.24
Occipital right	0.17	0.08	45.79	9.27	1.89	20.34	1.47	0.26	17.76
Occipital left	0.16	0.05	29.32	7.97	1.67	20.96	1.30	0.21	15.95
Parietal right	0.18	0.04	21.17	6.37	1.16	18.27	1.24	0.18	14.25
Parietal left	0.17	0.03	16.75	5.92	1.01	17.05	1.15	0.14	12.19
Peduncle right	0.28	0.04	14.32	4.69	0.65	13.80	1.27	0.17	13.37
Peduncle left	0.28	0.05	18.23	4.59	0.56	12.20	1.23	0.15	12.00
Pons right	0.45	0.11	24.90	3.94	0.90	22.73	1.74	0.42	24.08
Pons left	0.46	0.12	26.18	4.18	1.18	28.24	1.78	0.48	27.16
Temporal right	0.19	0.02	11.00	4.60	0.75	16.22	1.04	0.13	12.94
Temporal left	0.18	0.02	12.62	4.20	0.73	17.33	0.95	0.14	14.65
Mean	0.29	0.06	21.24	5.58	0.93	16.96	1.43	0.19	13.30

Intersubject variability for Choline/Creatine, N-Acetyl aspartate (NAA)/Choline and N-Acetyl aspartate/Creatine. Data displayed were obtained in 32 subjects and show mean, standard deviation (SD) and percentage coefficient of variation (CoV%) for each region of interest (ROI).

**Table 2:** Intersubject variability of metabolite concentrations for whole brain proton spectroscopy.

	NAA			Cho			Cr		
Region of Interest	Mean	SD	CoV %	Mean	SD	CoV %	Mean	SD	CoV %
Anterior corpus callosum	11778.3	2644.6	22.5	3928.9	1593.9	40.6	9561.7	3934.6	41.1
Body of corpus callosum	14215.7	1799.0	12.7	2535.7	1131.0	44.6	9357.7	2010.0	21.5
Posterior corpus callosum	14769.6	1782.9	12.1	2256.2	377.8	16.7	8508.9	1788.5	21.0
Corticospinal tract right	12802.3	765.3	6.0	2660.9	334.7	12.6	8612.0	561.5	6.5
Corticospinal tract left	12545.5	691.9	5.5	2651.9	407.9	15.4	8667.5	804.0	9.3
Anterior thalamic radiation right	11825.9	882.6	7.5	2973.8	1568.1	52.7	9130.7	3498.9	38.3
Anterior thalamic radiation left	12371.3	722.3	5.8	2958.6	1307.1	44.2	8946.8	1674.1	18.7
Inferior longitudinal fasciculus right	13292.0	1147.9	8.6	2220.6	817.6	36.8	9015.0	1466.9	16.3
Inferior longitudinal fasciculus left	12006.4	1119.4	9.3	1976.1	437.8	22.2	8232.2	794.6	9.7
Superior longitudinal fasciculus right	13347.6	992.9	7.4	2067.6	302.5	14.6	8578.7	613.6	7.2
Superior longitudinal fasciculus left	11969.7	1139.7	9.5	1905.6	285.7	15.0	7986.0	675.7	8.5
Thalamus right	12445.6	1926.6	15.5	2417.1	474.5	19.6	8043.5	1531.9	19.0
Thalamus left	12371.1	2000.5	16.2	2313.6	442.4	19.1	7825.0	1594.3	20.4
Pallidum right	12804.3	1312.2	10.2	2517.1	542.8	21.6	8952.5	1091.4	12.2
Pallidum left	13159.7	1388.3	10.5	2554.0	509.3	19.9	8877.8	1140.2	12.8
Putamen right	13276.7	1546.3	11.6	2601.2	647.9	24.9	9356.6	1079.3	11.5
Putamen left	13664.1	1426.8	10.4	2791.6	742.0	26.6	9473.1	1071.1	11.3
Dorsal Mid Brain	13584.3	2699.4	19.9	3003.7	791.2	26.3	8169.9	1740.5	21.3
Ventral Mid brain	13478.7	1588.7	11.8	2840.5	546.8	19.3	8576.1	1110.9	13.0
Frontal lobe right	7793.6	767.3	9.8	1991.8	1156.6	58.1	6519.3	2158.9	33.1
Frontal lobe left	7724.7	818.0	10.6	2042.6	1261.6	61.8	6379.4	1701.0	26.7
Hippocampus right	12640.0	1255.2	9.9	3121.1	928.0	29.7	9388.5	1823.7	19.4
Hippocampus left	12628.8	1315.5	10.4	3257.6	1660.3	51.0	9433.9	2998.5	31.8
Occipital right	12968.4	1817.8	14.0	1667.8	1146.9	68.8	8480.5	1905.3	22.5
Occipital left	11378.9	1562.1	13.7	1507.6	627.7	41.6	7655.5	1042.3	13.6
Parietal right	11321.4	1039.0	9.2	1668.9	532.8	31.9	7682.2	914.2	11.9
Parietal left	10300.9	977.7	9.5	1550.0	468.8	30.2	7164.8	834.4	11.6
Peduncle right	16640.8	1451.7	8.7	3771.9	618.9	16.4	13721.4	2252.4	16.4
Peduncle left	16388.9	1198.7	7.3	3760.7	927.0	24.6	13589.7	1511.0	11.1
Pons right	14199.4	2778.6	19.6	3757.8	918.3	24.4	8322.3	1989.1	23.9
Pons left	14712.8	2663.6	18.1	4076.3	1777.4	43.6	9308.0	5645.1	60.6
Temporal right	9137.6	1119.4	12.3	1834.7	341.9	18.6	6849.1	881.4	12.9
									
Temporal left	8266.0	1267.6	15.3	1683.3	344.8	20.5	6203.0	751.6	12.1
**Mean**	**12479.1**	**1442.7**	**11.6**	**2571.7**	**787.0**	**30.7**	**8683.9**	**1654.3**	**19.0**

Intersubject variability for N-Acetyl aspartate (NAA), Choline (Cho) and Creatine (Cr). Data displayed were obtained in 32 subjects and show mean, standard deviation (SD) and percentage coefficient of variation (CoV%) for each region of interest (ROI).

### Within session and between session reproducibility of WB ^1^HMRS

**Figure 3:**
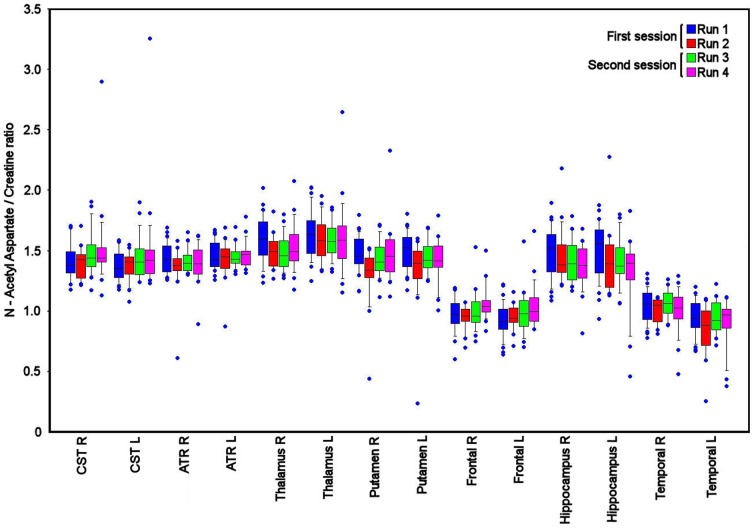
Variability in N Acetyl Aspartate/Creatine ratio measurements. Box and whisker plot for N Acetyl Aspartate/Creatine ratio for a selection of the regions of interest (ROI), including right (R) and left (L) corticospinal (CST), anterior thalamic radiation (ATR), thalamus, putamen, frontal lobe, hippocampus and temporal lobe. The spread of data within each ROI reflects inter subject variation, while the difference between runs 1 – 2 and 3 – 4 reflects within session reproducibility, and the change from first to second sessions reflects between session reproducibility. The central lines in each box denote median values, the lower and upper boundaries the 25th and 75th centile, the error bars the 10th and 90th centile, and the closed circles outlying data points.

**Figure 4:**
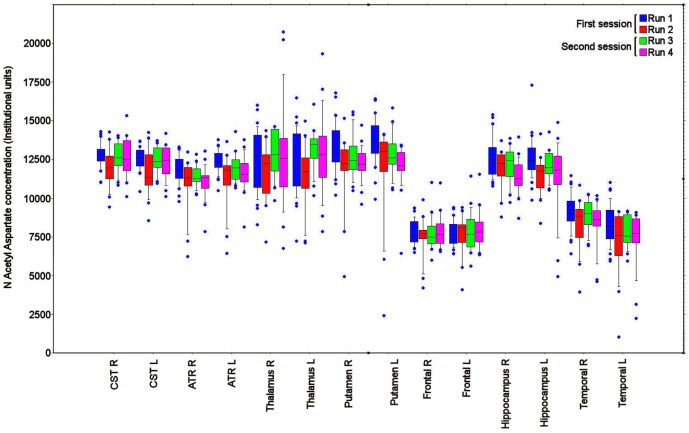
Variability in N Acetyl Aspartate concentration. Box and whisker plot for N Acetyl Aspartate for a selection of the regions of interest (ROI), including right (R) and left (L) corticospinal (CST), anterior thalamic radiation (ATR), thalamus, putamen, frontal lobe, hippocampus and temporal lobe. The spread of data within each ROI reflects inter subject variation, while the difference between runs 1 – 2 and 3 – 4 reflects within session reproducibility, and the change from first to second sessions reflects between session reproducibility. The central lines in each box denote median values, the lower and upper boundaries the 25th and 75th centile, the error bars the 10th and 90th centile, and the closed circles outlying data points.

The individual ROI data for within and between session reproducibility were variable across the different brain regions, but lower than the values for intersubject variability (Tables 3, 4, 5, 6). The within and between session reproducibility measurements were similar for Cho/Cr, NAA/Choline, Cho and Cr (11.8%, 11.4%, 14.3 and 10.6% *vs.* 11.9%, 11.4%, 13.5% and 10.5%, and p  =  0.44, 0.87, 0.08 and 0.86 respectively, paired ‘t’ tests), but for NAA/Creatine and NAA between session reproducibility was lower than within session reproducibility (9.3% and 9.1% *vs.* 10.1% and 9.9%, p <0.05 paired ‘t’ test with Bonferroni correction). The difference between intersubject variability, within and between session reproducibility is displayed for a selection of ROIs for the metabolite ratios and concentrations in Figs. 3 and 4 respectively.

**Table 3:** Within session and between session variability of metabolites for whole brain proton spectroscopy.

	Cho/Cr		NAA/Cho		NAA/Cr	
	Within Session	Between Session	Within Session	Between Session	Within Session	Between Session
**Anterior corpus callosum**	0.21 ± 0.21	0.23 ± 0.20	1.73 ± 1.40	2.63 ± 2.86	0.48 ± 0.42	0.50 ± 0.47
**Body of corpus callosum**	0.03 ± 0.03	0.05 ± 0.06	0.69 ± 0.65	0.68 ± 0.64	0.18 ± 0.16	0.16 ± 0.14
**Posterior corpus callosum**	0.04 ± 0.08	0.05 ± 0.08	0.51 ± 0.50	0.55 ± 0.56	0.25 ± 0.30	0.25 ± 0.31
**Corticospinal tract right**	0.03 ± 0.05	0.02 ± 0.03	0.52 ± 0.63	0.60 ± 0.70	0.09 ± 0.15	0.10 ± 0.15
**Corticospinal tract left**	0.03 ± 0.03	0.03 ± 0.05	0.48 ± 0.58	0.46 ± 0.55	0.11 ± 0.21	0.09 ± 0.17
**Anterior thalamic radiation right**	0.04 ± 0.08	0.05 ± 0.08	0.32 ± 0.37	0.32 ± 0.26	0.09 ± 0.13	0.06 ± 0.10
**Anterior thalamic radiation left**	0.04 ± 0.05	0.04 ± 0.04	0.29 ± 0.22	0.35 ± 0.33	0.06 ± 0.08	0.06 ± 0.06
**Inferior longitudinal fasciculus right**	0.03 ± 0.07	0.03 ± 0.09	0.50 ± 0.59	0.52 ± 0.48	0.07 ± 0.10	0.06 ± 0.09
**Inferior longitudinal fasciculus left**	0.02 ± 0.05	0.02 ± 0.04	0.50 ± 0.69	0.44 ± 0.53	0.08 ± 0.14	0.06 ± 0.09
**Superior longitudinal fasciculus right**	0.01 ± 0.02	0.01 ± 0.02	0.59 ± 0.74	0.61 ± 0.68	0.07 ± 0.07	0.06 ± 0.06
**Superior longitudinal fasciculus left**	0.01 ± 0.02	0.01 ± 0.01	0.38 ± 0.40	0.44 ± 0.34	0.06 ± 0.08	0.06 ± 0.06
**Thalamus right**	0.03 ± 0.03	0.03 ± 0.03	0.52 ± 0.45	0.46 ± 0.48	0.12 ± 0.12	0.12 ± 0.10
**Thalamus left**	0.03 ± 0.02	0.03 ± 0.03	0.56 ± 0.46	0.61 ± 0.51	0.14 ± 0.16	0.14 ± 0.13
**Pallidum right**	0.04 ± 0.04	0.03 ± 0.03	0.57 ± 0.66	0.51 ± 0.58	0.13 ± 0.17	0.12 ± 0.15
**Pallidum left**	0.05 ± 0.06	0.04 ± 0.05	1.11 ± 1.12	0.99 ± 0.95	0.18 ± 0.24	0.15 ± 0.17
**Putamen right**	0.03 ± 0.03	0.03 ± 0.03	0.66 ± 0.62	0.48 ± 0.52	0.12 ± 0.14	0.11 ± 0.13
**Putamen left**	0.04 ± 0.04	0.04 ± 0.04	0.74 ± 0.84	0.73 ± 0.80	0.14 ± 0.18	0.13 ± 0.15
**Dorsal mid brain**	0.04 ± 0.07	0.04 ± 0.07	0.46 ± 0.42	0.54 ± 0.45	0.27 ± 0.42	0.26 ± 0.40
**Ventral mid brain**	0.06 ± 0.06	0.05 ± 0.05	0.62 ± 0.58	0.55 ± 0.64	0.22 ± 0.32	0.20 ± 0.28
**Frontal lobe right**	0.04 ± 0.04	0.03 ± 0.04	0.47 ± 0.28	0.57 ± 0.48	0.07 ± 0.06	0.08 ± 0.08
**Frontal lobe left**	0.05 ± 0.05	0.04 ± 0.05	0.38 ± 0.31	0.50 ± 0.47	0.06 ± 0.05	0.07 ± 0.08
**Hippocampus right**	0.03 ± 0.03	0.02 ± 0.03	0.48 ± 0.33	0.49 ± 0.38	0.14 ± 0.11	0.13 ± 0.10
**Hippocampus left**	0.03 ± 0.04	0.03 ± 0.03	0.53 ± 0.48	0.52 ± 0.46	0.18 ± 0.15	0.16 ± 0.15
**Occipital right**	0.03 ± 0.08	0.05 ± 0.13	1.09 ± 1.09	1.22 ± 1.13	0.09 ± 0.13	0.11 ± 0.14
**Occipital left**	0.03 ± 0.07	0.03 ± 0.06	0.91 ± 0.92	0.90 ± 0.86	0.09 ± 0.10	0.09 ± 0.09
**Parietal right**	0.03 ± 0.08	0.03 ± 0.08	0.72 ± 0.68	0.81 ± 0.74	0.10 ± 0.13	0.12 ± 0.13
**Parietal left**	0.02 ± 0.04	0.02 ± 0.03	0.49 ± 0.50	0.69 ± 0.60	0.09 ± 0.14	0.11 ± 0.15
**Peduncle right**	0.10 ± 0.26	0.06 ± 0.19	0.43 ± 0.38	0.42 ± 0.38	0.17 ± 0.19	0.15 ± 0.17
**Peduncle left**	0.09 ± 0.33	0.08 ± 0.28	0.42 ± 0.39	0.39 ± 0.42	0.13 ± 0.22	0.12 ± 0.21
**Pons right**	0.06 ± 0.06	0.05 ± 0.04	0.63 ± 1.02	0.54 ± 0.79	0.21 ± 0.16	0.17 ± 0.15
**Pons left**	0.06 ± 0.06	0.06 ± 0.05	0.68 ± 1.23	0.53 ± 0.96	0.24 ± 0.19	0.22 ± 0.18
**Temporal right**	0.02 ± 0.04	0.02 ± 0.03	0.32 ± 0.33	0.39 ± 0.33	0.07 ± 0.08	0.05 ± 0.07
**Temporal left**	0.02 ± 0.02	0.02 ± 0.02	0.44 ± 0.55	0.39 ± 0.48	0.10 ± 0.13	0.08 ± 0.10
**Mean**	0.04 ± 0.10	0.04 ± 0.09	0.60 ± 0.73	0.63 ± 0.87	0.14 ± 0.20	0.13 ± 0.20

*Individual region of interest measurements for within session reproducibility obtained in the first and second imaging sessions in 17 and 16 subjects respectively, and the between session reproducibility for those 22 subjects who underwent imaging at both sessions. Data displayed are standard deviation for metabolite ratios (Choline (Cho)/Creatine (Cr), N-Acetyl aspartate (NAA)/Choline and NAA/Cr.*

**Table 4:** Within session and between session variability of metabolites for whole brain proton spectroscopy.

	Cho/Cr		NAA/Cho		NAA/Cr	
	Within Session	Between Session	Within Session	Between Session	Within Session	Between Session
**Anterior corpus callosum**	39.56 ± 34.49	46.44 ± 35.47	41.96 ± 40.19	47.03 ± 38.81	40.69 ± 40.56	38.17 ± 37.48
**Body of corpus callosum**	10.71 ± 7.72	14.59 ± 13.44	10.33 ± 9.86	10.53 ± 9.64	11.09 ± 9.58	9.92 ± 7.66
**Posterior corpus callosum**	10.36 ± 15.49	12.99 ± 15.49	7.78 ± 8.39	8.35 ± 8.72	12.93 ± 14.36	12.68 ± 14.41
**Corticospinal tract right**	8.15 ± 11.83	7.24 ± 8.49	8.92 ± 8.72	10.75 ± 11.00	5.57 ± 6.83	6.54 ± 7.48
**Corticospinal tract left**	8.21 ± 8.91	8.54 ± 11.98	8.63 ± 8.73	8.59 ± 8.94	6.50 ± 9.37	5.98 ± 7.99
**Anterior thalamic radiation right**	12.29 ± 17.17	12.49 ± 16.87	6.63 ± 8.65	6.34 ± 5.97	6.68 ± 11.84	4.62 ± 8.63
**Anterior thalamic radiation left**	11.74 ± 12.45	11.06 ± 11.21	5.39 ± 4.66	6.58 ± 6.58	4.45 ± 6.73	4.45 ± 4.72
**Inferior longitudinal fasciculus right**	7.47 ± 11.18	9.01 ± 14.85	7.70 ± 10.50	7.79 ± 8.22	5.17 ± 8.28	4.08 ± 6.72
**Inferior longitudinal fasciculus left**	8.80 ± 11.95	7.51 ± 9.58	8.80 ± 15.09	7.60 ± 11.26	6.50 ± 13.10	5.12 ± 9.11
**Superior longitudinal fasciculus right**	5.26 ± 7.34	5.46 ± 6.46	7.80 ± 8.51	8.47 ± 8.18	4.27 ± 3.98	3.94 ± 3.93
**Superior longitudinal fasciculus left**	5.99 ± 6.54	6.16 ± 5.52	6.32 ± 8.12	7.14 ± 6.22	4.37 ± 7.11	4.86 ± 5.35
**Thalamus right**	9.10 ± 7.04	9.05 ± 8.39	9.83 ± 8.49	8.38 ± 8.82	7.56 ± 7.23	7.91 ± 6.52
**Thalamus left**	8.44 ± 7.22	9.67 ± 8.10	10.16 ± 8.33	11.01 ± 9.07	8.81 ± 8.51	8.35 ± 7.58
**Pallidum right**	12.54 ± 12.38	10.84 ± 9.75	11.90 ± 18.78	9.93 ± 14.88	10.00 ± 16.35	8.58 ± 13.02
**Pallidum left**	14.69 ± 18.61	12.82 ± 15.19	20.59 ± 25.85	17.26 ± 20.01	14.13 ± 22.87	11.12 ± 16.82
**Putamen right**	11.28 ± 9.83	10.19 ± 10.22	13.04 ± 15.64	9.20 ± 12.42	8.98 ± 13.40	8.15 ± 11.15
**Putamen left**	11.96 ± 13.74	12.21 ± 13.27	14.48 ± 20.63	13.15 ± 16.67	11.21 ± 18.53	9.47 ± 14.13
**Dorsal mid brain**	10.58 ± 12.20	9.68 ± 11.80	9.89 ± 9.67	11.15 ± 9.24	14.38 ± 17.92	13.82 ± 15.09
**Ventral mid brain**	17.27 ± 16.76	14.07 ± 13.13	13.50 ± 17.99	10.99 ± 15.45	13.97 ± 21.91	12.09 ± 17.39
**Frontal lobe right**	16.12 ± 13.73	14.71 ± 15.11	10.03 ± 6.45	12.96 ± 11.93	6.84 ± 6.76	8.15 ± 8.28
**Frontal lobe left**	18.83 ± 15.03	17.00 ± 16.07	8.56 ± 7.40	11.34 ± 12.00	6.50 ± 5.81	7.34 ± 8.24
**Hippocampus right**	8.44 ± 9.05	7.71 ± 7.78	9.88 ± 6.36	9.92 ± 7.17	9.88 ± 8.01	9.03 ± 7.43
**Hippocampus left**	11.08 ± 15.17	8.87 ± 11.62	12.05 ± 12.26	11.51 ± 11.51	13.80 ± 13.84	11.98 ± 13.01
**Occipital right**	11.27 ± 15.53	13.04 ± 22.14	13.52 ± 17.83	13.83 ± 14.95	6.99 ± 12.66	7.68 ± 10.33
**Occipital left**	11.35 ± 18.54	11.08 ± 17.57	12.81 ± 15.86	12.21 ± 13.82	6.89 ± 9.52	7.13 ± 7.67
**Parietal right**	8.37 ± 13.84	10.15 ± 13.54	10.61 ± 9.97	12.15 ± 10.74	7.22 ± 8.23	9.06 ± 8.90
**Parietal left**	7.77 ± 12.53	9.27 ± 11.44	7.92 ± 8.51	11.36 ± 9.84	6.81 ± 8.96	9.02 ± 10.03
**Peduncle right**	16.93 ± 25.85	13.01 ± 19.52	9.75 ± 10.40	9.45 ± 9.10	12.55 ± 14.44	11.24 ± 11.89
**Peduncle left**	12.33 ± 21.86	12.20 ± 18.94	9.79 ± 10.34	8.91 ± 10.24	9.18 ± 10.44	8.93 ± 10.51
**Pons right**	12.89 ± 12.02	10.61 ± 8.31	13.10 ± 15.61	11.86 ± 13.39	12.72 ± 10.05	9.57 ± 9.16
**Pons left**	13.78 ± 12.87	14.85 ± 11.69	14.52 ± 15.40	11.69 ± 14.43	15.22 ± 12.94	12.61 ± 11.73
**Temporal right**	8.35 ± 12.58	8.46 ± 10.05	7.86 ± 9.41	9.07 ± 8.80	7.07 ± 9.77	5.63 ± 8.36
**Temporal left**	11.18 ± 13.93	9.34 ± 11.57	13.12 ± 20.92	10.86 ± 16.65	12.94 ± 18.93	9.63 ± 15.11
**Mean**	11.91 ± 15.77	11.83 ± 15.65	11.43 ± 15.62	11.44 ± 14.77	10.06 ± 15.29	9.30 ± 13.43

Individual region of interest measurements for within session reproducibility obtained in the first and second imaging sessions in 17 and 16 subjects respectively, and the between session reproducibility for those 22 subjects who underwent imaging at both sessions. Data displayed are percentage coefficient of variation for metabolite ratios (Choline (Cho)/Creatine (Cr), N-Acetyl aspartate (NAA)/Choline and NAA/Cr.

**Table 5:** Within session and between session variability of metabolites for whole brain proton spectroscopy.

	NAA		Cho		Cr	
	Within Session	Between Session	Within Session	Between Session	Within Session	Between Session
**Anterior corpus callosum**	3279.9 ± 2703.7	2968.5 ± 2697.1	1878.8 ± 2338.6	1719.7 ± 2458.7	3697.5 ± 2975.4	3814.5 ± 3818.6
**Body of corpus callosum**	1141.0 ± 815.6	1314.2 ± 1162.3	492.2 ± 1191.7	656.2 ± 1355.5	1201.2 ± 1391.4	1317.6 ± 1327.6
**Posterior corpus callosum**	1091.4 ± 989.5	1186.1 ± 1089.6	318.2 ± 376.5	323.3 ± 387.4	1309.3 ± 1398.6	1432.2 ± 1454.5
**Corticospinal tract right**	702.9 ± 734.4	590.2 ± 608.4	284.2 ± 646.5	256.6 ± 500.8	519.4 ± 671.9	504.0 ± 614.5
**Corticospinal tract left**	673.5 ± 700.7	558.0 ± 625.5	231.7 ± 240.5	196.4 ± 194.1	521.8 ± 544.5	378.7 ± 465.1
**Anterior thalamic radiation right**	827.2 ± 912.2	740.0 ± 775.1	422.3 ± 422.9	523.5 ± 598.9	716.5 ± 565.6	904.9 ± 1558.5
**Anterior thalamic radiation left**	789.7 ± 1023.5	727.1 ± 938.9	387.9 ± 374.1	424.0 ± 536.9	604.6 ± 677.1	731.0 ± 860.9
**Inferior longitudinal fasciculus right**	798.9 ± 929.8	816.7 ± 881.6	179.7 ± 175.5	287.8 ± 607.3	627.6 ± 553.7	712.5 ± 901.6
**Inferior longitudinal fasciculus left**	802.6 ± 1058.1	775.9 ± 969.3	207.5 ± 431.0	202.7 ± 317.7	419.8 ± 430.0	466.5 ± 498.6
**Superior longitudinal fasciculus right**	548.9 ± 655.5	624.2 ± 673.6	208.5 ± 285.7	216.2 ± 226.4	465.4 ± 459.7	471.0 ± 454.4
**Superior longitudinal fasciculus left**	623.6 ± 768.8	657.1 ± 726.0	197.0 ± 190.2	177.3 ± 178.5	493.8 ± 529.0	478.7 ± 508.6
**Thalamus right**	1307.2 ± 1442.7	1214.1 ± 1308.9	294.7 ± 242.3	266.5 ± 269.1	864.7 ± 682.2	968.4 ± 780.6
**Thalamus left**	1219.0 ± 1244.6	1301.8 ± 1171.3	330.9 ± 304.3	399.7 ± 438.0	996.5 ± 757.5	1079.0 ± 894.7
**Pallidum right**	1211.9 ± 1209.2	1087.1 ± 1259.3	427.3 ± 354.7	334.5 ± 299.4	847.8 ± 581.4	722.5 ± 635.1
**Pallidum left**	1457.9 ± 1760.3	1212.6 ± 1515.8	453.0 ± 300.8	365.3 ± 314.9	935.8 ± 814.1	829.6 ± 789.2
**Putamen right**	1154.7 ± 1145.8	1084.7 ± 1280.7	368.4 ± 404.2	317.3 ± 407.0	794.9 ± 720.1	770.7 ± 726.4
**Putamen left**	1286.2 ± 1634.9	1289.1 ± 1553.6	416.5 ± 365.3	413.6 ± 434.6	804.7 ± 763.3	775.7 ± 788.2
**Dorsal Mid Brain**	2179.9 ± 2135.5	1996.1 ± 1847.3	423.3 ± 328.8	415.8 ± 311.8	1160.2 ± 1060.2	1229.0 ± 881.1
**Ventral Midbrain**	1348.3 ± 1791.0	1184.9 ± 1597.5	412.7 ± 360.1	316.6 ± 315.8	947.6 ± 939.3	901.7 ± 835.9
**Frontal lobe right**	451.9 ± 529.9	548.9 ± 575.8	379.3 ± 499.0	409.2 ± 396.5	700.7 ± 667.7	756.8 ± 912.2
**Frontal lobe left**	516.0 ± 575.2	596.0 ± 565.1	537.9 ± 560.2	430.3 ± 463.8	826.1 ± 826.4	739.7 ± 591.6
**Hippocampus right**	861.7 ± 658.3	845.6 ± 764.6	388.7 ± 341.1	289.6 ± 226.4	797.4 ± 555.3	676.4 ± 532.2
**Hippocampus left**	1050.0 ± 1232.9	1001.4 ± 1147.3	380.9 ± 300.4	338.5 ± 278.2	853.3 ± 765.6	790.2 ± 659.6
**Occipital right**	658.8 ± 781.0	749.0 ± 837.7	235.3 ± 368.6	390.9 ± 986.0	526.1 ± 653.5	770.4 ± 1264.1
**Occipital left**	582.0 ± 799.6	567.2 ± 692.2	277.4 ± 611.4	269.8 ± 644.9	496.8 ± 542.5	545.5 ± 717.1
**Parietal right**	528.1 ± 648.3	536.3 ± 609.6	232.4 ± 445.5	208.5 ± 334.0	465.2 ± 594.3	494.0 ± 540.5
**Parietal left**	507.5 ± 598.6	527.3 ± 610.9	177.0 ± 216.8	152.8 ± 192.4	487.6 ± 581.7	491.7 ± 535.7
**Peduncle right**	1578.9 ± 1632.0	1470.6 ± 1444.0	770.9 ± 1507.8	541.6 ± 1107.7	1199.7 ± 1218.9	1295.7 ± 1422.0
**Peduncle left**	1357.3 ± 1592.7	1337.8 ± 1269.4	960.6 ± 3271.4	862.4 ± 2572.0	1065.0 ± 1330.2	1230.5 ± 1142.6
**Pons right**	1590.4 ± 1560.1	1617.1 ± 1589.4	411.3 ± 295.7	395.4 ± 384.1	990.4 ± 638.2	1048.3 ± 776.8
**Pons left**	1569.6 ± 1464.3	1401.8 ± 1561.0	553.4 ± 612.4	368.8 ± 474.5	1532.3 ± 2594.0	985.7 ± 797.7
**Temporal right**	843.6 ± 924.7	768.5 ± 884.4	185.3 ± 137.6	176.5 ± 204.3	513.8 ± 503.8	557.1 ± 551.7
**Temporal left**	892.9 ± 1170.0	839.5 ± 1017.6	196.4 ± 159.4	183.8 ± 192.8	545.4 ± 674.7	522.7 ± 591.2
**Mean**	**1073.7** ± **1350.1**	**1034.4** ± **1280.9**	**412.8** ± **896.0**	**388.8** ± **835.9**	**876.6** ± **1163.6**	**890.7** ± **1223.2**

*Individual region of interest measurements for within session reproducibility obtained in the first and second imaging sessions in 17 and 16 subjects respectively, and the between session reproducibility for those 22 subjects who underwent imaging at both sessions. Data displayed are standard deviation) for metabolite concentrations (NAA, Cho and Cr).*

**Table 6:** Within session and between session variability of metabolites for whole brain proton spectroscopy.

	NAA		Cho		Cr	
	Within Session	Between Session	Within Session	Between Session	Within Session	Between Session
**Anterior corpus callosum**	38.0 ± 41.5	33.1 ± 38.1	44.6 ± 37.8	40.5 ± 37.9	42.1 ± 38.2	37.5 ± 37.2
**Body of corpus callosum**	8.6 ± 6.2	9.5 ± 8.4	15.8 ± 17.8	18.8 ± 22.3	13.7 ± 14.0	14.6 ± 13.9
**Posterior corpus callosum**	8.0 ± 7.4	8.4 ± 7.8	13.9 ± 13.7	13.9 ± 14.7	16.1 ± 15.7	17.6 ± 16.9
**Corticospinal tract right**	5.7 ± 6.2	4.8 ± 5.2	8.8 ± 13.6	8.1 ± 11.1	6.1 ± 7.5	5.9 ± 7.0
**Corticospinal tract left**	5.7 ± 6.3	4.7 ± 5.6	8.7 ± 8.3	7.6 ± 7.3	6.4 ± 7.9	4.7 ± 6.7
**Anterior thalamic radiation right**	7.9 ± 9.7	6.9 ± 8.1	14.5 ± 12.9	16.9 ± 14.8	8.5 ± 7.0	9.5 ± 10.4
**Anterior thalamic radiation left**	7.2 ± 10.4	6.5 ± 9.1	13.0 ± 10.7	14.7 ± 15.6	7.0 ± 7.6	8.4 ± 9.6
**Inferior longitudinal fasciculus right**	6.6 ± 8.2	6.6 ± 7.5	8.3 ± 7.0	9.6 ± 13.1	7.0 ± 5.8	7.6 ± 7.8
**Inferior longitudinal fasciculus left**	7.6 ± 11.5	7.1 ± 10.1	8.5 ± 11.2	8.7 ± 9.4	5.4 ± 6.1	5.8 ± 6.5
**Superior longitudinal fasciculus right**	4.4 ± 5.8	5.0 ± 5.8	10.1 ± 11.7	10.5 ± 10.0	5.6 ± 6.1	5.8 ± 6.1
**Superior longitudinal fasciculus left**	5.5 ± 7.7	5.8 ± 7.2	10.4 ± 9.5	9.5 ± 9.3	6.5 ± 8.3	6.4 ± 7.9
**Thalamus right**	10.5 ± 11.0	9.9 ± 9.8	12.5 ± 10.6	11.6 ± 11.5	10.7 ± 9.0	12.3 ± 10.4
**Thalamus left**	10.2 ± 11.5	10.9 ± 10.3	14.3 ± 13.0	16.7 ± 16.5	12.7 ± 10.9	13.7 ± 11.8
**Pallidum right**	11.1 ± 14.4	9.6 ± 13.2	16.7 ± 13.3	13.1 ± 12.3	10.1 ± 7.8	8.8 ± 9.1
**Pallidum left**	14.3 ± 23.4	11.0 ± 18.4	19.3 ± 15.3	15.5 ± 14.9	12.5 ± 16.6	10.6 ± 14.1
**Putamen right**	10.0 ± 12.0	9.0 ± 11.9	14.2 ± 13.0	12.3 ± 14.0	9.1 ± 8.6	8.9 ± 9.1
**Putamen left**	12.1 ± 19.4	11.1 ± 16.4	16.6 ± 14.3	16.0 ± 16.0	10.0 ± 12.3	9.3 ± 12.0
**Dorsal Mid Brain**	15.5 ± 15.3	14.2 ± 12.1	14.0 ± 11.9	14.1 ± 10.7	13.2 ± 12.1	14.8 ± 10.8
**Ventral Midbrain**	13.0 ± 24.1	10.3 ± 18.6	16.4 ± 16.1	12.5 ± 13.7	12.9 ± 16.3	11.8 ± 13.4
**Frontal lobe right**	6.3 ± 8.6	7.6 ± 9.0	18.3 ± 16.7	20.8 ± 17.4	11.0 ± 10.1	11.6 ± 11.8
**Frontal lobe left**	7.1 ± 9.0	8.1 ± 8.6	23.4 ± 16.2	20.7 ± 19.7	12.3 ± 10.9	11.7 ± 9.7
**Hippocampus right**	7.4 ± 5.9	7.1 ± 6.6	13.0 ± 8.6	10.5 ± 8.1	8.7 ± 5.9	7.8 ± 6.3
**Hippocampus left**	9.6 ± 12.8	8.7 ± 11.4	12.9 ± 10.4	12.1 ± 10.0	9.1 ± 7.5	9.0 ± 7.7
**Occipital right**	5.6 ± 7.5	6.1 ± 7.1	11.4 ± 14.1	13.0 ± 18.8	5.9 ± 7.3	7.7 ± 9.8
**Occipital left**	5.6 ± 8.9	5.4 ± 7.9	11.3 ± 16.0	11.4 ± 15.7	6.1 ± 6.7	6.6 ± 7.4
**Parietal right**	4.7 ± 5.9	4.8 ± 5.5	10.3 ± 11.9	10.0 ± 10.7	5.8 ± 7.6	6.4 ± 7.2
**Parietal left**	4.8 ± 5.7	5.1 ± 5.8	9.7 ± 8.8	8.5 ± 8.5	6.6 ± 8.6	6.9 ± 7.9
**Peduncle right**	10.9 ± 13.0	9.8 ± 11.0	15.9 ± 19.1	12.0 ± 15.5	10.1 ± 12.7	9.9 ± 11.5
**Peduncle left**	9.8 ± 13.3	9.2 ± 10.3	13.3 ± 22.2	12.3 ± 18.7	8.9 ± 14.0	9.7 ± 11.5
**Pons right**	13.5 ± 16.4	12.1 ± 13.2	12.7 ± 11.4	11.7 ± 11.8	13.4 ± 9.6	13.4 ± 10.4
**Pons left**	12.9 ± 14.9	10.5 ± 13.0	14.5 ± 16.7	11.1 ± 15.5	15.7 ± 16.7	12.7 ± 11.4
**Temporal right**	10.8 ± 13.5	9.2 ± 12.1	10.6 ± 7.4	9.5 ± 9.0	8.5 ± 9.2	8.6 ± 8.8
**Temporal left**	14.4 ± 24.0	12.0 ± 19.1	13.6 ± 14.3	12.1 ± 14.0	11.2 ± 17.4	10.0 ± 14.4
**Mean**	**9.9** ± **15.4**	**9.1** ± **13.4**	**14.3** ± **15.9**	**13.5** ± **16.2**	**10.6** ± **13.8**	**10.5** ± **13.2**

*Individual region of interest measurements for within session reproducibility obtained in the first and second imaging sessions in 17 and 16 subjects respectively, and the between session reproducibility for those 22 subjects who underwent imaging at both sessions. Data displayed are percentage coefficient of variation for metabolite concentrations (NAA, Cho and Cr).*

The intraclass correlation coefficient (ICC) for within and between session reproducibility within brain regions of mixed cortical and deep grey, and white matter are displayed in table 7.

**Table 7:** Within session and between session intraclass correlation coefficient for metabolites.

	Mixed		White	
	Within	Between	Within	Between
**Cho/Cr**	0.76(0.72 − 0.80)	0.71(0.68 − 0.75)	0.50(0.41 − 0.58)	0.58(0.53 − 0.63)
**NAA/Cho**	0.84(0.82 − 0.87)	0.82(0.80 − 0.84)	0.78(0.74 − 0.82)	0.56(0.51 − 0.61)
**NAA/Cr**	0.76(0.72 − 0.80)	0.79(0.76 − 0.81)	0.60(0.52 − 0.66)	0.55(0.49 − 0.60)
**NAA**	0.81(0.77 − 0.84)	0.80(0.78 − 0.83)	0.63(0.56 − 0.68)	0.58(0.53 − 0.63)
**Cho**	0.84(0.81 − 0.86)	0.75(0.71 − 0.78)	0.53(0.44 − 0.60)	0.61(0.56 − 0.66)
**Cr**	0.84(0.81 − 0.86)	0.77(0.74 − 0.79)	0.73(0.68 − 0.77)	0.66(0.61 − 0.70)

Data displayed are mean (95% Confidence interval) intraclass correlation coefficient for metabolite ratios (Choline (Cho)/Creatine (Cr), N Acetyl Aspartate (NAA)/Cho, NAA/Cr) and metabolites (NAA, Cho and Cr) for mixed cortical and deep grey, and white matter brain regions.

### Calculation of 95% prediction interval for zero change

Using the four WB ^1^HMRS measurements obtained from both sessions we used ANOVA to determine the significance of the differences (Tables 8 and 9). These confirm that there is a significant difference between regions and subjects, and that there is a significant interaction between brain region and subject. The residual variance of the measurements that cannot be accounted for by the known independent variables is shown in Tables 8 and 9. The calculated SD values were 0.10, 1.03 and 0.28 for Cho/Cr, NAA/Cho, NAA/Cr and 1709.7, 913.2 and 1521.4 iu for NAA, Cho and Cr respectively. The overall population 95% prediction interval for zero change (based on two SD values) were therefore 0.20, 2.06 and 0.56 for Cho/Cr, NAA/Cho and NAA/Cr and 3419.4, 1826.4 and 3042.8 iu for NAA, Cho and Cr respectively. For the within session measurements the calculated SD values were 0.10, 1.11 and 0.23 for Cho/Cr, NAA/Cho, NAA/Cr and 1399.7, 1115.9 and 1292.8 iu for NAA, Cho and Cr respectively and were similar to the data obtained from all four sessions. These data can be used to calculate prediction intervals within individual ROIs. For the within session data (Table 3 & 5) an estimate of the 95% prediction intervals for zero change within individual ROIs should be based on 4.3 SD values. As an example, this results in a 95% prediction interval for zero change for NAA, Cho and Cr within a single imaging session of 3839.5, 844.5 and 2345.2 iu for the left temporal, and 3557.0, 1815.9 and 3081.0 iu for the right anterior thalamic radiation respectively. These prediction intervals can be used to assess the impact of therapeutic interventions within a single session, but also to assess the impact of treatment and disease progression over time within different imaging sessions.

**Table 8:** Analysis of variance table for metabolite ratios.

Parameter	Session	DF	Sum of Squares	Mean Square	F Value	p Value
**Cho/Cr**	ROI	32	17.18	0.54	52.86	<.0001
	subject	31	2.86	0.09	9.07	<.0001
	ROI * subject	992	14.70	0.01	1.46	<.0001
	Residual	1815	18.44	0.01		
**NAA/Cho**	ROI	32	3105.79	97.06	91.19	<.0001
	subject	31	657.96	21.22	19.94	<.0001
	ROI * subject	992	1264.47	1.27	1.2	0.0006
	Residual	1815	1931.76	1.06		
**NAA/Cr**	ROI	32	113.60	3.55	61.42	<.0001
	subject	31	24.16	0.78	13.48	<.0001
	ROI * subject	992	83.45	0.08	1.46	<.0001
	Residual	1815	104.90	0.08		

Data were obtained from 32 volunteers using the region of interest (ROI) template for metabolite ratios. (Choline – Cho, Creatine – Cr, N acetyl aspartate – NAA and DF – Degrees of freedom).

**Table 9:** Analysis of variance table for metabolite ratios.

Parameter	Session	DF	Sum of Squares	Mean Square	F Value	p Value
**NAA**	ROI	32	8.3 × 10^9^	2.6 × 10^8^	89.1	<.0001
	subject	31	1.2 × 10^9^	3.7 × 10^7^	12.8	<.0001
	ROI * subject	992	3.7 × 10^9^	3.7 × 10^6^	1.3	<.0001
	Residual	1815	5.3 × 10^9^	2.9 × 10^6^		
**Cho**	ROI	32	1.2 × 10^9^	3.7 × 10^7^	43.9	<.0001
	subject	31	3.5 × 10^8^	1.1 × 10^7^	13.4	<.0001
	ROI * subject	992	1.5 × 10^9^	1.5 × 10^6^	1.9	<.0001
	Residual	1815	1.5 × 10^9^	8.3 × 10^5^		
**Cr**	ROI	32	4.7 × 10^9^	1.5 × 10^8^	63.3	<.0001
	subject	31	1.0 × 10^9^	3.3 × 10^7^	14.1	<.0001
	ROI * subject	992	4.4 × 10^9^	4.4 × 10^6^	1.9	<.0001
	Residual	1815	4.2 × 10^9^	2.3 × 10^6^		

Data were obtained from 32 volunteers using the region of interest (ROI) template for and metabolite concentrations. (Choline – Cho, Creatine – Cr, N acetyl aspartate – NAA and DF – Degrees of freedom).

## Discussion

This study provides additional reference data concerning intersubject variability and reproducibility of metabolite ratios and individual signal-intensity normalised metabolite concentrations obtained using WB ^1^HMRS conducted within the same imaging session (within session) and different imaging sessions (between session) in a group of healthy volunteers. As reported previously, we found that intersubject variability was high [21]. The reproducibility of metabolite ratios and concentrations were lower than intersubject variability (10 – 15% *vs.* 15 – 30%) but there was substantial variability across the brain for all the calculated parameters. The within and between session reproducibility measurements were similar for Cho/Cr, NAA/Cho, Cho and Cr but for NAA/Creatine and NAA between session reproducibility was lower than within session reproducibility. The calculated overall population 95% prediction intervals for zero change of repeat MIDAS measurements were 0.20, 2.06 and 0.56 for metabolite ratios (Cho/Cr, NAA/Cho and NAA/Cr) and 3419.4, 1826.4 and 3042.8 iu for metabolite concentrations (NAA, Cho and Cr) respectively. These prediction intervals can be calculated for individual ROIs and utilised in interventional studies where response to therapy can be assessed, or to assess the significance of change from disease progression within longitudinal studies of nervous system disorders.

The factors affecting the reproducibility of WB ^1^HMRS parameters include changes within the MR scanner or individual subjects. Features related to the scanner include B_0_ field inhomogeneities (heating during the long acquisition process), scanner drift, gradient coil stability, signal to noise ratio and software upgrades. Such factors may be more significant when imaging is acquired within different imaging sessions, rather than repeat acquisitions within the same session where such parameters are more likely to be similar. Regular servicing and daily quality assurance measurements seek to ensure that an MR scanner is operating normally. It is obviously necessary to monitor such changes, and where possible, take steps to limit their impact on the spectroscopic data obtained. Importantly, there were no upgrades or changes in MR scanner hardware or software during the period of this study. While scanner variability is important there are individual subject factors that can induce substantial variability in WB ^1^HMRS. These include head movements and positioning within the scanner field of view. In particular, data acquisition within the volume of interest is sensitive to inhomogeneities that can result from proximity to the sphenoid and frontal sinuses. We undertook standard procedures to limit such variability. All subjects were positioned within the head coil according to standard operating procedures within our institution and the alignment confirmed prior to commencing imaging. Following standard imaging for localisation we monitored subject movement, and all data were checked during processing for movement artefact. No data sets were excluded in these analyses due to subject motion during the scan. In addition, we performed all analyses following image coregistration and spatial normalisation to MNI standard space. We used a standard ROI template covering the whole brain from the Harvard Oxford subcortical and MNI structural probabilistic atlases available within FSL. While the use of this analysis strategy sought to reduce variability within our comparisons, we eroded the ROI template by a single voxel within FSL in order to improve spatial localisation and reduce the impact of coregistration, normalisation and partial volume errors. Finally, all ROIs were manually inspected to ensure that they were correctly aligned with the imaging data and corresponded to the regions specified. In summary, we considered possible sources of WB ^1^HMRS variability within our centre and attempted to limit their impact and ensure that the data we acquired were comparable within and between the different imaging sessions.

Whilst our results for WB ^1^HMRS reproducibility are in line with published data, we report data specifically concerning the difference between intersubject variability, within session and between session reproducibility. It is useful to consider the sources of variability in WB ^1^HMRS data in the setting where we are trying to address the significance of changes between normal physiology and disease states, or changes that are the consequence of a therapeutic intervention. In the first case, the relevant sources of error are the intersubject variability in the patient and volunteer groups. Our data for healthy volunteers are broadly concordant with results from other groups [24], and show that these are high, with mean (range) CoV for Cho/Cr 21 (11 – 62%), NAA/Cho 17 (11 – 55%), NAA/Cr 13 (8 – 37%), NAA 12 (6 – 23%), Cho 31 (13 – 69%) and Cr 19 (7 – 61%). To be certain that ^1^HMRS values derived from an individual patient are significantly lower, with a confidence of 95%, these figures suggest that we need to have mean ROI NAA values (for example) that are at least 23% lower than volunteer means. This estimate and the secure distinction of a patient group as abnormal is confounded by the fact that intersubject CoV in patients with neurological disorders is likely to be larger than controls, and variable across different brain regions. These figures underline the difficulty of using WB ^1^HMRS in small groups of patients with different causes of neurological disease who have variable pathophysiology. In practice, the estimated study sample size is moderated by the dramatic changes in metabolite concentration that occur in patients. For example, following mild traumatic brain injury there is approximately a 20% reduction in NAA and increase in Cho even where structural imaging appears normal, and in severe traumatic brain injury changes of up to a 50% can occur [35], [36]. Hence the significance of metabolite change is often detected with manageable numbers, despite the large intersubject variability in volunteer and patients groups.

However, it is important to point out that these figures are largely irrelevant when considering the power and design of clinical studies, when WB ^1^HMRS is being used to monitor changes within the same subject in the same scanning session (within session reproducibility) or during longitudinal assessments over time in several different imaging sessions (between session reproducibility). In such settings, the subject is his or her own control, and the relevant parameter is intrasubject variability or reproducibility. Our data show that these figures for CoV are smaller than those obtained from the discussion in the previous paragraph. In addition, we provide reference data for metabolites in healthy volunteers demonstrating that the CoV for within session reproducibility is broadly comparable to that obtained in different imaging sessions (Tables 3, 4, 5, 6, 7). While the reproducibility of NAA/Creatine and NAA was significantly lower for between session compared to within session measurements the absolute differences were small. This finding is not consistent with the lack of difference for the other metabolites and is unlikely to be clinically relevant. We found no evidence to suggest that within session reproducibility was smaller than between session reproducibility measurements. These data provide helpful guidance for designing clinical studies, and suggest that for NAA or NAA/Cr it should be possible to detect differences of 20% with confidence. For example, although the reproducibility of measurements is variable for the different brain regions we can use these data to calculate sample sizes for interventional and longitudinal clinical studies. For a lobar ROI such as the right frontal region the between session CoV was 8% for NAA and we should be able to detect a 20% change with 95% power at a significance level of 1% within a group of 10 subjects within a single interventional or longitudinal study design [37]. Clearly, such estimates only strictly apply to our scanner and institution, but they provide a useful starting point for any spectroscopic study design. There are a number of factors particular to our scanning protocols and institutional setup that limit the use of the reproducibility measurements that we provide. These include, but might not be limited to, scanner, acquisition protocols, data correction and reconstruction, and processing. Despite these variations, it should be possible for other groups to use the methodology that we describe to derive ‘in house’ data for their studies. In addition, although these data provide guidance for designing clinical studies, particular groups of subjects (including those with brain injury) may require sedation and control of ventilation as part of clinical care. While such patient groups may appear complex and difficult to manage within the context of an imaging study the fact that they remain completely immobile and have stable physiology should result in lower CoV for reproducibility measurements and an increase in the sensitivity of interventional studies[34].

### Methodological limitations

The volunteers included in this study ranged in aged from 25 – 50 years, and since metabolite levels are associated with age [23], this may account for some of the variability in the intersubject analysis. While we were able to obtain multiple WB ^1^HMRS datasets on up to two occasions in this group of volunteers, scanner availability and subject tolerance (duration and noise) prevented us from acquiring further WB ^1^HMRS datasets within the same session and additional scanning sessions. A repeat imaging session was performed within a mean (range) of 33 (3 – 181) days, and variation in this interval could result in biological differences between the datasets obtained within a few days compared to those obtained after several months. However, any expected change in WB ^1^HMRS in healthy volunteers of a similar age over a period of up to six months is small and unlikely to have resulted in the differences we have found [23], [38]. In addition, we found no relationship between scan reproducibility and the interval between the two imaging sessions.

We found variability in the ^1^HMRS measurements and their reproducibility across the different brain regions. In addition, there was more variability in metabolite data involving choline, which probably reflects the lower concentration of choline within the brain [39]. These differences are demonstrated in Tables 1, 2, 3, 4, 5, 6, and Figs. 3 and 4 and were particularly relevant for the corpus callosum, deep grey matter, midbrain, frontal, occipital and some white matter regions. We found no relationship between the ROI volume and intersubject variability and reproducibility of ^1^HMRS for any of the metabolites (data not shown). Despite this, the cause of these differences may in part be related to inhomogeneities in the B_0_ field induced by the frontal and sphenoidal air sinuses, partial volume errors within relatively small regions, locally variant metabolite concentrations, and variation in the quality of coregistration and spatial normalisation within individual subjects. We tried to limit these errors through careful review of all the transformed imaging datasets, shimming the scanner before each MIDAS data acquisition, and eroding the ROI template by a single voxel to improve accuracy. Despite this, errors remain within some ROIs where ^1^HMRS values differ in closely adjacent brain regions. However, the purpose of this study was to determine the variability of measurements using an ROI template and standard processing pipeline. While variability in the fitting of template ROIs in individual subjects may result in higher intersubject variability for particular brain regions this should be less likely for measurements of reproducibility within the same subject. Here any differences in ROI template fitting between the sessions should be small. These regional differences underline that ^1^HMRS studies should compare data within the same brain region using the same data processing technique. Our figures for reproducibility are higher than that reported by Maudsley et al using the same acquisition sequence [20]. This reflects our inclusion of a larger study group and that we utilised a standard processing pipeline and ROI template covering the whole brain within normalised space that we would typically apply to patient studies. While the data we report are specific to our methods the reproducibility measurements that we report provide a useful starting point for study design.

## Conclusions

This study provides additional reference data concerning intersubject variability and reproducibility of WB ^1^HMRS conducted in a group of healthy volunteers. The CoV for repeat WB ^1^HMRS measurements obtained during the same session were similar to that obtained from measurements obtained in a different imaging session separated by up to six months. These data can be used to calculate the 95% prediction interval for zero change and may inform the design of interventional studies to quantify change within a single imaging session, or to assess the significance of change in longitudinal studies.

## References

[1] Price SJ, Gillard JH (2011) Imaging biomarkers of brain tumour margin and tumour invasion. Br J Radiol 84 Spec No 2:S159–S167. 22433826 10.1259/bjr/26838774 PMC3473903

[2] Govind V, Gold S, Kaliannan K, Saigal G, Falcone S, et al (2010) Whole-Brain Proton MR Spectroscopic Imaging of Mild-to-Moderate Traumatic Brain Injury and Correlation with Neuropsychological Deficits. J Neurotrauma 27:483–496. 20201668 10.1089/neu.2009.1159 PMC2867627

[3] Govindaraju V, Gauger GE, Manley GT, Ebel A, Meeker M, et al (2004) Volumetric proton spectroscopic imaging of mild traumatic brain injury. AJNR Am J Neuroradiol 25:730–737. 15140711 PMC7974501

[4] Holshouser BA, Tong KA, Ashwal S (2005) Proton MR spectroscopic imaging depicts diffuse axonal injury in children with traumatic brain injury. AJNR Am J Neuroradiol 26:1276–1285. 15891197 PMC8158612

[5] Gonen O, Catalaa I, Babb JS, Ge Y, Mannon LJ, et al (2000) Total brain N-acetylaspartate: a new measure of disease load in MS. Neurology 54:15–19. 10636119 10.1212/wnl.54.1.15

[6] Ruiz-Peña JL, Piñero P, Sellers G, Argente J, Casado A, et al (2004) Magnetic resonance spectroscopy of normal appearing white matter in early relapsing-remitting multiple sclerosis: correlations between disability and spectroscopy. BMC Neurol 4:8. 15191618 10.1186/1471-2377-4-8 PMC446197

[7] Govind V, Sharma KR, Maudsley AA, Arheart KL, Saigal G, et al (2012) Comprehensive evaluation of corticospinal tract metabolites in amyotrophic lateral sclerosis using whole-brain 1H MR spectroscopy. PloS one 7:e35607. 22539984 10.1371/journal.pone.0035607 PMC3335096

[8] Watanabe T, Shiino A, Akiguchi I (2012) Hippocampal metabolites and memory performances in patients with amnestic mild cognitive impairment and Alzheimer's disease. Neurobiol Learn Mem 97:289–293. 22390859 10.1016/j.nlm.2012.01.006

[9] Kraguljac NV, Reid M, White D, Jones R, den Hollander J, et al (2012) Neurometabolites in schizophrenia and bipolar disorder - a systematic review and meta-analysis. Psychiatry Res 203:111–125. 22981426 10.1016/j.pscychresns.2012.02.003 PMC3466386

[10] Szulc A, Waszkiewicz N, Bibulowicz D, Konarzewska B, Tarasow E (2012) Proton magnetic resonance spectroscopy changes after antipsychotic treatment. Curr Med Chem 23157634

[11] Steen RG, Hamer RM, Lieberman JA (2005) Measurement of brain metabolites by 1H magnetic resonance spectroscopy in patients with schizophrenia:a systematic review and meta-analysis. Neuropsychopharmacology 30:1949–1962. 16123764 10.1038/sj.npp.1300850

[12] Moffett JR, Ross B, Arun P, Madhavarao CN, Namboodiri AMA (2007) N-Acetylaspartate in the CNS: from neurodiagnostics to neurobiology. Prog Neurobiol 81:89–131. 17275978 10.1016/j.pneurobio.2006.12.003 PMC1919520

[13] Klein J (2000) Membrane breakdown in acute and chronic neurodegeneration: focus on choline-containing phospholipids. J Neural Transm 107:1027–1063. 11041281 10.1007/s007020070051

[14] Tumati S, Martens S, Aleman A (2013) Magnetic resonance spectroscopy in mild cognitive impairment: Systematic review and meta-analysis. Neurosci Biobehav Rev 10.1016/j.neubiorev.2013.08.004 23969177

[15] Miller BL, Chang L, Booth R, Ernst T, Cornford M, et al (1996) In vivo 1H MRS choline: correlation with in vitro chemistry/histology. Life Sci 58:1929–1935. 8637421 10.1016/0024-3205(96)00182-8

[16] Wylezinska M, Cifelli A, Jezzard P, Palace J, Alecci M, et al (2003) Thalamic neurodegeneration in relapsing-remitting multiple sclerosis. Neurology 60:1949–1954. 12821738 10.1212/01.wnl.0000069464.22267.95

[17] Maudsley AA, Darkazanli A, Alger JR, Hall LO, Schuff N, et al (2006) Comprehensive processing, display and analysis for in vivo MR spectroscopic imaging. NMR Biomed 19:492–503. 16763967 10.1002/nbm.1025 PMC2673915

[18] Ebel A, Soher BJ, Maudsley AA (2001) Assessment of 3D proton MR echo-planar spectroscopic imaging using automated spectral analysis. Magn Reson Med 46:1072–1078. 11746571 10.1002/mrm.1301

[19] Maudsley AA, Wu Z, Meyerhoff DJ, Weiner MW (1994) Automated processing for proton spectroscopic imaging using water reference deconvolution. Magn Reson Med 31:589–595. 8057811 10.1002/mrm.1910310603

[20] Maudsley AA, Domenig C, Sheriff S (2010) Reproducibility of serial whole-brain MR spectroscopic imaging. NMR Biomed 23:251–256. 19777506 10.1002/nbm.1445 PMC2917802

[21] Maudsley AA, Domenig C, Govind V, Darkazanli A, Studholme C, et al (2009) Mapping of brain metabolite distributions by volumetric proton MR spectroscopic imaging. Magn Reson Med 61:548–559 (MRSI). 19111009 10.1002/mrm.21875 PMC2724718

[22] Langer DL, Rakaric P, Kirilova A, Jaffray DA, Damyanovich AZ (2007) Assessment of metabolite quantitation reproducibility in serial 3D-(1)H-MR spectroscopic imaging of human brain using stereotactic repositioning. Magn Reson Med 58:666–673. 17899591 10.1002/mrm.21351

[23] Maudsley AA, Govind V, Arheart KL (2012) Associations of age, gender and body mass with 1H MR-observed brain metabolites and tissue distributions. NMR Biomed 25:580–593. 21858879 10.1002/nbm.1775 PMC3313016

[24] Li BSY, Babb JS, Soher BJ, Maudsley AA, Gonen O (2002) Reproducibility of 3D proton spectroscopy in the human brain. Magn Reson Med 47:439–446. 11870829 10.1002/mrm.10081

[25] Veenith TV, Carter E, Grossac J, Newcombe VFJ, Outtrim JG, et al (2013) Inter subject variability and reproducibility of diffusion tensor imaging within and between different imaging sessions. PloS one 8:e65941. 23840380 10.1371/journal.pone.0065941 PMC3696006

[26] Ebel A, Maudsley AA (2001) Comparison of methods for reduction of lipid contamination for in vivo proton MR spectroscopic imaging of the brain. Magn Reson Med 46:706–712. 11590647 10.1002/mrm.1249

[27] Woolrich MW, Jbabdi S, Patenaude B, Chappell M, Makni S, et al (2009) Bayesian analysis of neuroimaging data in FSL. Neuroimage 45:S173–S186. 19059349 10.1016/j.neuroimage.2008.10.055

[28] Greve DN, Fischl B (2009) Accurate and robust brain image alignment using boundary-based registration. Neuroimage 48:63–72. 19573611 10.1016/j.neuroimage.2009.06.060 PMC2733527

[29] Jenkinson M, Smith S (2001) A global optimisation method for robust affine registration of brain images. Med Image Anal 5:143–156. 11516708 10.1016/s1361-8415(01)00036-6

[30] Jenkinson M, Bannister P, Brady M, Smith S (2002) Improved optimization for the robust and accurate linear registration and motion correction of brain images. Neuroimage 17:825–841. 12377157 10.1016/s1053-8119(02)91132-8

[31] Klein A, Andersson J, Ardekani BA, Ashburner J, Avants B, et al (2009) Evaluation of 14 nonlinear deformation algorithms applied to human brain MRI registration. Neuroimage 46:786–802. 19195496 10.1016/j.neuroimage.2008.12.037 PMC2747506

[32] Coles JP, Fryer TD, Bradley PG, Nortje J, Smielewski P, et al (2006) Intersubject variability and reproducibility of 15O PET studies. J Cereb Blood Flow Metab 26:48–57. 15988475 10.1038/sj.jcbfm.9600179

[33] Coles JP, Fryer TD, Coleman MR, Smielewski P, Gupta AK, et al (2007) Hyperventilation following head injury:effect on ischemic burden and cerebral oxidative metabolism. Crit Care Med 35:568–578. 17205016 10.1097/01.CCM.0000254066.37187.88

[34] Coles JP, Fryer TD, Bradley PG, Nortje J, Smielewski P, et al (2006) Intersubject variability and reproducibility of 15O PET studies. J Cereb Blood Flow Metab 26:48–57. 15988475 10.1038/sj.jcbfm.9600179

[35] Vagnozzi R, Signoretti S, Cristofori L, Alessandrini F, Floris R, et al (2010) Assessment of metabolic brain damage and recovery following mild traumatic brain injury:a multicentre, proton magnetic resonance spectroscopic study in concussed patients. Brain 133:3232–3242. 20736189 10.1093/brain/awq200

[36] Signoretti S, Marmarou A, Aygok GA, Fatouros PP, Portella G, et al (2008) Assessment of mitochondrial impairment in traumatic brain injury using high-resolution proton magnetic resonance spectroscopy. J Neurosurg 108:42–52. 18173309 10.3171/JNS/2008/108/01/0042

[37] Faul F, Erdfelder E, Lang AG, Buchner A (2007) G*Power 3:a flexible statistical power analysis program for the social, behavioral, and biomedical sciences. Behav Res Methods 39:175–191. 17695343 10.3758/bf03193146

[38] Saunders DE, Howe FA, van den Boogaart A, Griffiths JR, Brown MM (1999) Aging of the adult human brain:in vivo quantitation of metabolite content with proton magnetic resonance spectroscopy. J Magn Reson Imaging 9:711–716. 10331768 10.1002/(sici)1522-2586(199905)9:5<711::aid-jmri14>3.0.co;2-3

[39] Christiansen P, Toft P, Larsson HB, Stubgaard M, Henriksen O (1993) The concentration of N-acetyl aspartate, creatine + phosphocreatine, and choline in different parts of the brain in adulthood and senium. Magn Reson Imaging 11:799–806. 8371635 10.1016/0730-725x(93)90197-l

